# Evaluation of Left Atrial Function in Patients with Paroxysmal Atrial Fibrillation Using Left Atrial Automatic Myocardial Functional Imaging Ultrasonography

**DOI:** 10.1155/2023/6924570

**Published:** 2023-11-20

**Authors:** Hailan Liu, Lili Chen, Yan Song, Yingying Xu, Chunquan Zhang

**Affiliations:** ^1^Department of Ultrasound, Nanchang First Hospital, Nanchang 330006, Jiangxi, China; ^2^Department of Ultrasound, The Second Affiliated Hospital of Nanchang University, Nanchang, Jiangxi 330006, China

## Abstract

**Aim:**

To evaluate volume and strain of the left atrium (LA) in people suffering from paroxysmal atrial fibrillation which is not valvular (NVPAF) using the new technology of left atrial automatic myocardial function imaging (AFILA) and to analyze prognostic factors in patients with NVPAF by follow-up.

**Methods:**

Between August 2019 and August 2022, a total of 80 NVPAF patients and 60 normal control patients who were hospitalized in the Department of Cardiology were included in the study. The LA volume and strain parameters of the two groups were analyzed. The differences in LA function (LAF) parameters were compared between the two groups to generate the receiver operating characteristic curve (ROC) and calculate the area under the curve (AUC), sensitivity, and specificity of each parameter. Follow-up was conducted on the 80 NVPAF patients included, their treatment methods after admission and their rehospitalization due to heart events were recorded, and independent risk factors influencing the prognosis of NVPAF were obtained.

**Results:**

A total of 140 patients participated in the study, including 80 in the NVPAF group and 60 in the normal control group. There was no statistically significant difference in age and sex between the two groups. Compared to the normal group, the LA minimum volume (LAVmin), LA maximum volume (LAVmax), and volume at onset of LA contraction (LAVpreA) in the NVPAF group were significantly increased. The LA emptying fraction (LAEF) was significantly decreased, and LA reservoir strain (S_R), LA conduit strain (S_CD), and LA contractile strain (S_CT) were significantly compromised (*P* < 0.05). There was no significant difference in LA evacuation volume (LAEV) reduction (*P* > 0.05). Logistic regression analysis of LAF parameters in NVPAF patients showed that LAEF and S_R were independently correlated with NVPAF (odds ratio values: 0.883 (0.827–0.943), *P* < 0.001; 0.916 (0.569–1.474), *P* = 0.047). The ROC curve results showed that LAEF had a high efficiency in the diagnosis of NVPAF, with *P* < 0.001, AUC of 0.843, sensitivity of 0.788, and specificity of 0.867. For the LA strain parameters, the S_R test efficiency was higher, with *P* < 0.001, AUC of 0.762, sensitivity of 0.713, and specificity of 0.783. There was a strong correlation between S_R and LAEF in patients with no end event and those with end event. The ROC curve revealed that the S_R was better than LAEF in predicting prognosis of patients with AF (AUC = 0.914, *P* < 0.0001 vs. AUC = 0.876, *P* < 0.0001). S_R of 10.5 and LAEF of 21 were the cut-off values for endpoint events in NVPAF patients, with sensitivity of 0.909 and 0.727 and specificity of 0.904 and 0.901, respectively.

**Conclusions:**

AFILA ultrasound technology comprehensively evaluated the LA size and function in patients with NVPAF. The LAEF and S_R were independently correlated with NVPAF and can determine the prognosis of patients with NVPAF.

## 1. Introduction

Atrial fibrillation (AF) is a common cardiovascular disease. At present, there are at least 10 million AF patients in China, and this number increases every year. It is one of the most studied arrhythmias [1–4] that accounts for one-fifth of stroke events and is currently recognized as an independent risk factor for stroke [1, 5]. AF can be categorized into paroxysmal AF (PAF), persistent AF, and permanent AF [6]. Among them, PAF is characterized by an AF duration of ≤7 days (usually ≤2 days), which can spontaneously cease and begin again. Compared to cerebral infarction caused by non-AF, AF-related stroke has a significantly higher risk of disability or death [7, 8]. The left atrium (LA) of patients with PAF is enlarged and fibrotic, leading to a serious decline in its function [9], which plays an important role in the onset, maintenance, and progression of AF [10, 11]. Therefore, accurate evaluation of LAF has important clinical significance. Ultrasound has been recognized as a noninvasive method to evaluate LA structure and function, for which there are many measurement methods. The commonly used techniques include tissue Doppler imaging, spot tracking technology, and real-time three-dimensional echocardiography measurement. All of them measure the LA strain and strain rate to determine the LAF via myocardial strain analysis. Because most of the above methods use left ventricular software to analyze LA, some challenges are inevitable. The anatomical structure of the left ventricle is different from that of the LA. The thin wall, complex course of LA muscle fibers, foramen ovale on the atrial septum, and four openings of the pulmonary veins on the LA wall all affect the accuracy and repeatability of LA strain measurement results [12, 13].

The 4DAutoLAQ is the new ultrasound technology specially used for the measurement of LAF. It computes the three-dimensional volume data and the LA strain using a semiautomatic segmentation approach. The strain and LA volume parameters are automatically obtained by the program, but excellent image quality is necessary for this technique. When the LA envelope is incomplete during image acquisition or when the image quality is low, the analysis findings vary significantly.

An innovative technique for assessing LAF is automatic left atrial function imaging (AFILA). Spot tracking is used on two-dimensional echocardiography to estimate global strain and LA volume. The LA volume/strain-time curve, LA volume, emptying fraction, and other parameters can all be obtained by the operator with ease and speed. It can also be used in combination with three planes during the onset of PAF and persistent AF. Compared to the 4DAutoLAQ software, its operation is simple. The method also has good repeatability and is less affected by image quality.

## 2. Methods

### 2.1. Study Population

Participants in the study were 60 normal controls and 80 patients with nonvalvular paroxysmal atrial fibrillation (NVPAF) who were admitted to the Department of Cardiology at the Second Affiliated Hospital of Nanchang University between January 2019 and April 2022. There were 50 males and 30 females in the NVPAF group, with an average age of 61.59 ± 9.4 years. There were 35 males and 25 females in the normal control group, with an average age of 58.8 ± 5.2 years. The hospital ethics committee approved this trial, and each patient provided informed permission.

### 2.2. Inclusion/Exclusion Criteria

The inclusion criteria for the NVPAF group are as follows: (1) patients who were confirmed to have PAF by an electrocardiogram (ECG), (2) patients who were diagnosed with nonvalvular AF using echocardiography, and (3) patients with clear ultrasonic images. The exclusion criteria are as follows: (1) persistent and permanent AF, (2) combined rheumatic mitral stenosis, mitral valvuloplasty and repair, and prosthetic valve replacement, (3) primary myocardial and pericardial disease, (4) congenital heart disease, and (5) incomplete medical records. Inclusion and exclusion criteria for the control group were the same as for the NVPAF group except for the presence of sinus rhythm as inclusion criteria and history of arrythmia as exclusion criteria.

### 2.3. Image Acquisition

Imaging was done using the GE vivid E95 ultrasonic diagnostic instrument (GE Healthcare; Vingmed Ultrasound, Horten, Norway) outfitted with an M5S probe (frequency: 1.5–4.6 MHz) and EchoPAC 204 software (GE Healthcare). The apical four- and two-chamber images with >5 cardiac cycles were collected with the imaging frame rate of >40 fps after confirming that the 2D-section standard was stable. Software analysis was performed on the saved dynamic clear images.

### 2.4. AFILA Analysis

**Figure 1:**
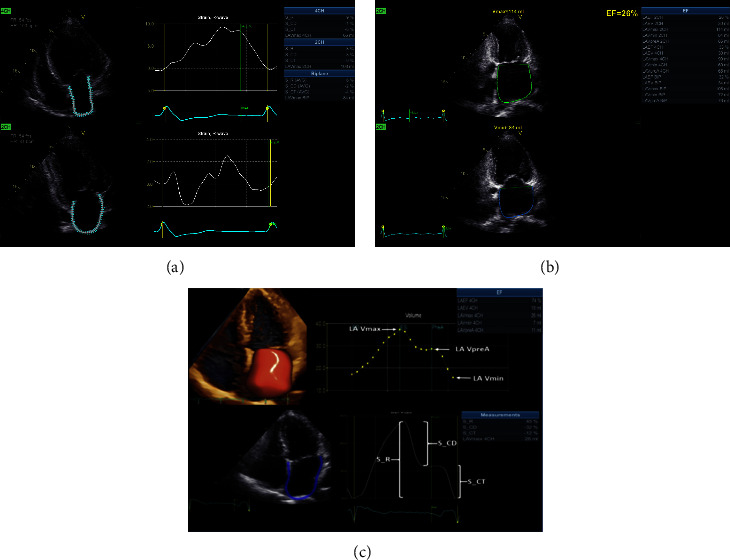
(a) AFILA obtained left atrial strain results of patients with paroxysmal atrial fibrillation with four-chamber heart and two-chamber heart. (b) Left atrial volume parameters of four-chamber and two-chamber heart obtained by AFILA in patients with paroxysmal atrial fibrillation. (c) Left atrial volume and parameters of patients in the normal group obtained by AFILA.

The software EchoPAC 204 was used to import the images. The apical four-chamber view and two-chamber views were used to make sure that the sampling point was positioned in the septal base, lateral wall base, lower wall base, and LA apex as indicated in the top right corner after clicking “measure” and choosing AFILA to enter the measurement mode. After a short wait of a few seconds, the LA volume and strain parameters of the four-chamber view and two-chamber view were obtained. The biplane's LA parameter average was also noted (Figures 1(a)–1(c)).

Volume characteristics include left atrial emptying fraction (LAEF), left atrial minimum volume (LAVmin), left atrial maximum volume (LAVmax), left atrial evacuation volume (LAEV), and volume at the beginning of left atrial contraction (LAVpreA).

LA strain parameters consist of the following: left atrial contractile strain (S_CT), left atrial conduit strain (S_CD), and left atrial reservoir strain (S_R). As the LA wall became longer throughout the reservoir phase, the S_R Parameter is expressed as a positive number during this time. The S_CD and S_CT parameters in the other two phases had negative values because the LA wall shortened during these phases (Figure 1).

### 2.5. Follow-Up Analysis

Follow-up was conducted on 80 NVPAF patients included, recording their treatment methods after admission and their rehospitalization due to heart events. Telephone inquiries and inquiries from our hospital's information system were the main methods, and follow-up was conducted every three months. The starting time for follow-up is the time of receiving echocardiography examination, and the endpoint time was December 30, 2022. Acute heart failure attack, psychogenic syncope, stroke, recurrence of atrial fibrillation with rapid ventricular rate, acute coronary syndrome, additional arrhythmias, and death were the designated endpoint events. Endpoint events and nonendpoint events were the two categories into which the cases in the follow-up research were separated. Endpoint events were defined as one or more outcomes that were contingent upon the occurrence of endpoint events. 389 days was the median follow-up period.

### 2.6. Statistical Analysis

The statistical analysis was performed with IBM Corp.'s SPASS23.0 program (Chicago, IL, USA). The mean ± standard deviation was used to express the measurement data that were subject to normal distribution, and a *t*-test was used to conduct the intergroup comparison. For non-normal distribution, the measurement data were reported as the median with a 25–75% interquartile range, and a nonparametric test was used to compare the groups. In order to derive the AFILA parameters for independent risk ultrasound for PAF, the multivariate logistic regression analysis incorporated the indexes for intergroup comparison *P* value of <0.05. Kaplan-Meier method and comparisons between curves were assessed by the log-rank test.Cox regression analysis was performed to screen the independent factors that can predict the prognosis of atrial fibrillation patients. The participants' receiver operating characteristic curve (ROC) was created, and the sensitivity and specificity of each parameter as well as the area under the curve (AUC) were determined. The parameter value corresponding to the Youden index with maximum points (Youden index = sensitivity + specificity-1) was delineated by the ROC. *P* < 0.05 values were regarded as statistically significant. The AFILA parameters' consistency was assessed using the intragroup correlation coefficient (ICC). The ICC values ranged from 0 to 1. For ICC values between 0.75 and 0.9, the consistency was good; for ICC values more than 0.9, it was exceptional.

## 3. Results

### 3.1. General Data Comparison

The NVPAF group included of 80 patients, with an average age of 61.59 ± 9.4 years, comprising 50 males and 30 females. The study included 60 normal controls, with an average age of 58.8 ± 5.2 years; of these, 35 were male and 25 were female. Age and sex did not significantly differ between the two groups (*P* > 0.05). The level of mitral regurgitation, body mass index (BMI), hypertension, diabetes, hyperlipidemia, and left ventricular ejection fraction (LVEF) did not greatly differ between the two groups (*P* > 0.05) (Table 1).

### 3.2. LAF Parameter Comparison Using AFILA Ultrasound

The LA volume parameters LAVmin, LAVmax, and LAVpreA in the NVPAF group were significantly increased compared to those in the control group. In addition, LAEF was significantly decreased and LA strain parameters (S_R, S_CD, and S_CT) were significantly compromised (*P* < 0.05) (Table 2).

### 3.3. Logistic Regression Analysis of LAF Parameters in NVPAF Patients

In order to avoid the interaction between LA volume and strain parameters, the parameters with statistically significant differences in single factor analysis in Table 1 are evaluated using multifactor analysis. The results showed that LAEF and S_ R were independently correlated with NVPAF (odds ratio (OR) values: 0.883 (0.827–0.943), *P* = 0.000; 0.916 (0.569–1.474), *P* = 0.047) (Table 3).

### 3.4. Test Efficacy for PAF LA Volume and Strain Parameters

**Figure 2:**
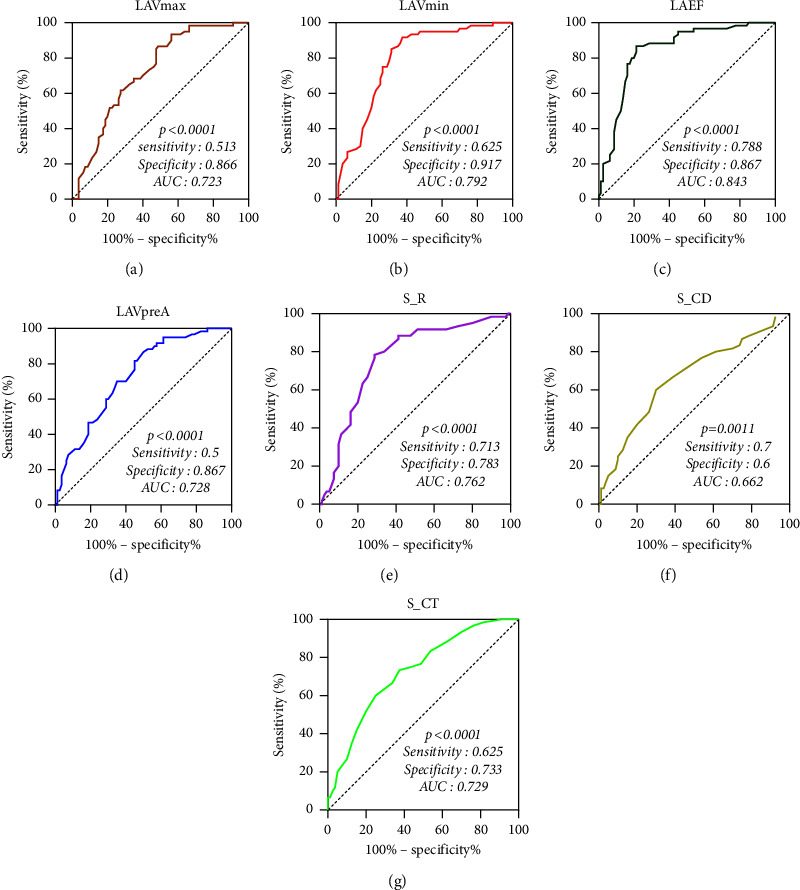
Comparison of ROC curves of left atrial volume and strain parameters in patients with atrial fibrillation. (a) The ROC curve of LAVmax (left atrial maximum volume) test for atrial fibrillation; (b) the ROC curve of LAVmin (left atrial minimum volume) test for atrial fibrillation; (c) the ROC curve of LAEF (left atrial emptying fraction) to test atrial fibrillation; (d) the ROC curve of LAVPreA (left atrial volume before systole) test for atrial fibrillation; (e) the ROC curve of S_ R (left atrial reservoir period strain) test for atrial fibrillation; (f) the ROC curve of S_CD (left atrial conduit strain) for testing atrial fibrillation; (g) the ROC curve of S_CT (left atrial contractile strain) test for atrial fibrillation.

As can be seen from the above results, there existed a significant difference (*P* < 0.05) among LAVmin, LAVmax, LAVpreA, LAEF, S_R, S_CD, and S_CT in the NVPAF group compared to the normal group. Seven indexes were used as test variables, and PAF was used as a state variable. The ROC curves were generated to ascertain the test efficacy for every index. The results showed that LAEF had a high test efficiency in the diagnosis of PAF (*P* < 0.001, AUC of 0.843, sensitivity of 0.788, and specificity of 0.867). The AUC values for the remaining LA volume parameters LAVmin, LAVmax, and LAVpreA were 0.792, 0.723, and 0.728, respectively. The sensitivity values were 0.625, 0.513, and 0.50, respectively. The specificity values were 0.917, 0.866, and 0.867, respectively (*P* < 0.0001). Among the LA strain parameters, the S_R had a higher test efficiency, with *P* < 0.0001, AUC of 0.762, sensitivity of 0.713, and specificity of 0.783. The AUC values for the remaining LA strain parameters S_CT and S_CD were 0.729 and 0.662, respectively, with the respective sensitivity of 0.625 and 0.7 and specificity of 0.733 and 0.6 (*P* < 0.0001 and 0.0011, respectively). The efficacy of LAEV in detecting PAF was not tested because there was no statistical difference in LAEV between the NVPAF and normal groups (Figure 2).

### 3.5. Consistency Test on Ultrasonic AFILA Measurement Parameters

Twenty patients were chosen at random for interobserver variability, and the same operator measured and examined the LA strain and volume parameters of AFILA in each patient over a period of time using the same methodology. Furthermore, the identical technique for measurement and analysis was used by another operator who met the same prerequisites. The determination of intraobserver variability was achieved by the comparison of data among various operators. All of the ICC values were >0.9, which denotes very good consistency (Table 4).

### 3.6. Follow-Up Analysis Results

**Figure 3:**
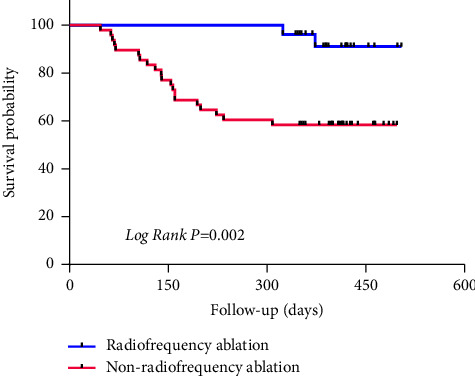
Survival analysis of atrial fibrillation treatment.

**Figure 4:**
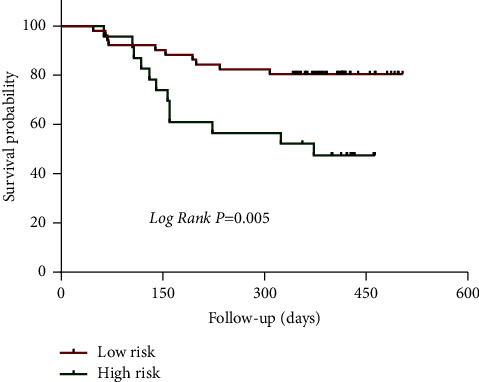
Thromboembolic risk survival analysis.

**Figure 5:**
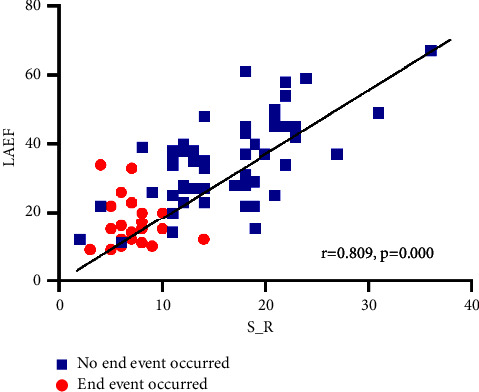
Bivariate correlation between S_R and LAEF for patients without end events and with end events. S_R, left atrial reservoir strain; LAEF, left atrial emptying fraction.

**Figure 6:**
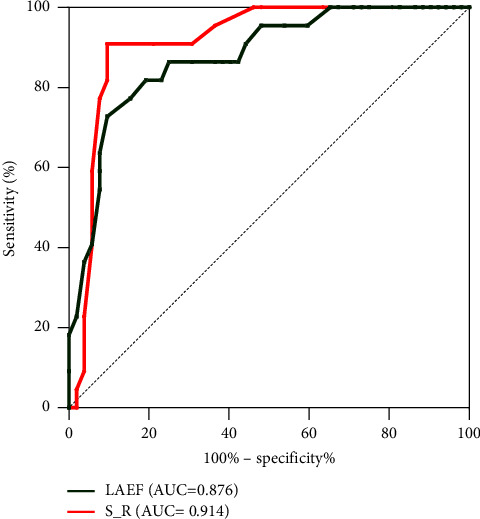
S_R, LAEF ROC curve for predicting the prognosis of atrial fibrillation; ROC, receiver operating characteristic; AUC, area under the curve; S_R, left atrial reservoir strain; LAEF, left atrial emptying fraction.

80 NVPAF patients were followed up, with 6 patients lost and the remaining 74 patients. Following up for 1-2 years, 25 patients were treated with radiofrequency ablation, 20 patients had cerebral infarction (including 5 cases after radiofrequency therapy), 13 patients were admitted due to recurrence of AF with rapid ventricular rate, 5 patients were admitted due to heart failure, 2 patients were admitted due to syncope, and 1 patient died. Univariate analysis found that treatment methods for AF, risk of thromboembolism in AF, S_ R, and LAEF were related to the prognosis of patients with AF, as shown in Tables 5 and 6. The survival of the radiofrequency ablation group was better than that of the nonradiofrequency ablation group (median survival time of 490 vs. 349 days, *P* = 0.002), the survival of the low risk group was better than that of the high risk group (median survival time of 434 vs. 310 days, *P* = 0.005) (see Figures 3 and 4). Multiple factor Cox regression analysis found that S_ R and LAEF were independently associated with the prognosis of AF, but not with the risk of thromboembolism or the treatment of AF, as shown in Table 5. There was a strong correlation between S_R and LAEF in patients with no end event and those with end event (Figure 5). The ROC curve revealed that the S_R was better than LAEF in predicting prognosis of patients with AF (AUC = 0.914, *P* < 0.0001 vs. AUC = 0.876, *P* < 0.0001). Further calculation shows that the maximum Jordan indices were 0.813 and 0.631, respectively, with corresponding values of 10.5 and 21. S_ R of 10.5 and LAEF of 21 were the cut-off values for endpoint events in NVPAF patients, with sensitivity of 0.909 and 0.727 and specificity of 0.904 and 0.901, respectively (Figure 6).

## 4. Discussion

PAF is a common arrhythmia in clinical practice. Many patients with PAF have no symptoms at all, or their arrhythmia spontaneously ceases immediately before an ECG examination and remains undetected. AF can result in a number of deadly and severe consequences, including cardiac failure and stroke, which can be avoided or managed with medicine. Thus, it is very important to identify and timely diagnose patients with PAF who present with sinus rhythm. AF may also lead to structural LA fibrosis, modifications in electric ion channels, atrial remodeling, and other changes [14]. The development and prevalence of PAF are tightly correlated with aberrant LAF. LA enlargement and fibrosis are symptoms of AF and are crucial in the creation, maintenance, and advancement of AF. [15]. The level of LA fibrosis can be assessed using delayed enhanced cardiac magnetic resonance imaging, but its application in routine clinical practice is confined because it is expensive, operationally complex, and time-consuming. It is currently believed that fibrosis is related to LA remodeling and compliance reduction. Therefore, LAF parameters evaluated by echocardiography can be considered to be a substitute for LA fibrosis [16]. AFILA is a new method to evaluate LAF using spot tracking on two-dimensional echocardiography. The software automatically obtains LA volume and strain results. Due to the absence of a P wave in patients with AF, the QRS complex should be used as the zero baseline and ventricular end diastole as the zero reference value, according to the EACVI/EHRA experts [17]. The maximum positive longitudinal strain defines the atrial storage function (S_R). The early and late diastolic strains define the atrial conduit (S_CD) and atrial systolic pump (S_CT) functions, respectively. The present study found that the volume/strain-time curve for each cardiac cycle in the normal group was in the shape of a “double peak and double valley,” with the highest peak at LAVmax, additional peak at LVPreA, and lowest point at LAVmin. This curve was steep (Figure 1(c)). In patients with PAF, the LA volume increases and the strain is damaged. The volume/strain-time curve for the LA is generally in the shape of a “single peak and double valley,” and its highest and lowest points are LAVmax and LAVmin, respectively. The LVPreA peak is not obvious or it might disappear. The volume/strain rate curve for the LA is flat, sometimes presenting with “multiple peaks and multiple valleys.” The LA storage function was damaged during left ventricular systole, while the LA conduit function and systolic pump function were damaged or even lost in the early and late diastole (Figure 1(a)). Multiple peaks and valleys were sometimes present. The volume parameters LAVmax, LAVmin, and LVPreA in PAF patients increased, and the LAEF decreased significantly, demonstrating the relationship between PAF and LA volume increase and LAF damage. Multifactor analysis showed that S_ R, LAEF, and PAF were independently related. LAEF reflected the LA storage function, and the storage period strain (S_R) test for the LA strain parameters was more effective, indicating that the study results were consistent. These results were also consistent with the results described by Jiang et al. [18, 19].

Compared to the normal group in the present study, the LA volume in NVPAF patients increased and the volume-time curve was flat, which was independently related to the LA S_R damage. Chen et al. [20] believed that the LA systolic strain in patients with AF was independently correlated. This inconsistency may be present because most patients in previous studies had persistent AF, which is different from PAF. Another reason was that the variables were different when included in the multivariate analysis. The present study compared the normal and stroke groups from the past. Providência et al. [21] have shown that the greater the LA volume, the higher the risk of AF, especially when the mitral valve was closed, and the higher the specificity of LAVmin. The volume parameter was LAEF in the ROC curve analysis, while the strain parameter was S_R. These two indicators were more sensitive than others when testing for AF, indicating that the LA reservoir strain and emptying function in patients with AF were further damaged. LA strain is a promising index. Habibi M et al. [22] have found that the LA strain and strain rate measured by speckle tracking echocardiography were significantly correlated with atrial muscle fibrosis, but not with the measured LA size.

In subsequent follow-up studies, we found that the risk of thromboembolism defined by the CHA2DS2-VASc score in survival analysis univariate studies, whether NVPAF received radiofrequency ablation treatment, and the values of S_ R and LAEF are all correlated with the prognosis of NVPAF patients, but in multivariate COX regression, we found that S_ R and LAEF are independent influencing factor for predicting the prognosis of NVPAF patients. The risk of thromboembolism and treatment methods defined by the CHA2DS2-VASc score cannot predict the prognosis of NVPAF patients. The possible reason is that the prognostic endpoint events of AF in this article include acute heart failure attack, AF recurrence with rapid ventricular rate, cardiogenic syncope, stroke, acute coronary syndrome, other arrhythmias, and death. The former has a significant correlation with the prognosis of stroke, while the latter has a significant correlation with the prognosis of recurrent AF.

AFILA ultrasound technology is a newly developed method based on the principle of two-dimensional spot tracking. It can directly obtain the volume/strain-time curve for the LA using corresponding software and quickly and easily identify the relevant parameters of the LA volume and strain [23]. Compared to the new technology of three and four-dimensional ultrasound LA evaluation, it has the advantages of extremely simple operation, high accuracy, good repeatability, and fast speed. It can be used for clinical routine development rather than be limited to scientific research. For all I know, this study is the first to apply this technique to analyze the relationship between LA volume and strain in PAF. However, the present study had limitations because it did not perform a comparative analysis of other AF types. In addition, the study sample size was small, making it necessary to expand it in order to obtain more objective results in the future. The risk of thromboembolism and treatment methods defined by the CHA2DS2-VASc score cannot predict the prognosis of NVPAF patients. The possible reason is that the prognostic endpoint events of atrial fibrillation in this article include acute heart failure attack, atrial fibrillation recurrence with rapid ventricular rate, cardiogenic syncope, stroke, acute coronary syndrome, other arrhythmias, and numerous factors of death. The former has a significant correlation with the prognosis of stroke, while the latter has a significant correlation with the prognosis of recurrent atrial fibrillation.

## 5. Conclusion

The present study showed that the LA volume and strain parameters in NVPAF patients were significantly different from those in the normal group. AFILA had a high sensitivity and specificity when evaluating the LAF of patients with PAF. The LA S_R and LAEF were shown to be independently related to PAF. Therefore, the LA volume and strain parameters could be used to accurately identify PAF to guide clinical practice. AFILA technology was simple and fast and could be used as a routine method for cardiac ultrasound examination.

## Figures and Tables

**Table 1:** Comparison of baseline data between control group and NVPAF group.

Clinical characteristics	Control (*n* = 60)	NVPAF (*n* = 80)	*P* value
Male gender, *n* (%)	35 (58.3)	50 (62.5)	0.203
Age (years) (mean ± SD)	58.8 ± 5.2	61.59 ± 9.4	0.420
LVEF (%) (mean ± SD)	60.48 ± 6.89	58.70 ± 8.23	0.176
BMI (kg/m^2^) (mean ± SD)	22.69 ± 2.77	23.57 ± 2.58	0.561
Hypertension, *n* (%)	19 (31.7)	38 (47.5)	0.059
Diabetes, *n* (%)	6 (10.0)	15 (18.8)	0.151
Hyperlipidemia, *n* (%)	8 (13.3)	13 (16.3)	0.632

Mitral regurgitation, *n* (%)			
None	10 (16.7)	7 (11.7)	0.099
Mild	40 (66.6)	58 (72.5)	
Moderate	10 (16.7)	15 (18.8)	
Severe	0	0	

Mean ± SD, the values are presented as the mean ± standard deviation; the remaining values represent the number and proportion of patients. NVPAF, nonvalvular paroxysmal atrial fibrillation; BMI, body mass index; LVEF, left ventricular ejection fraction.

**Table 2:** Comparison of left atrial volume and strain parameters between NVPAF group and normal control group.

LA function	NVPAF	Control	*Z*/*t*	*P*
LAVmin	44 (22.5∼62)	19 (14∼27.5)	−5.895	0.000
LAVmax	64.5 (45∼86)	42 (34∼56.75)	−4.506	0.000
LAVpreA	50.5 (34∼71)	34 (23.25∼45)	−4.617	0.000
LAEV	20 (15∼26.75)	23 (18∼29)	−2.052	0.057
LAEF	35 ± 14.84	55.5 (51∼60.75)	−6.924	0.000
S_R	11 (6∼18.75)	21 (17∼26.75)	−5.300	0.000
S_CD	−8 (−12∼−3.25)	−11 (−15.75∼−8)	−3.273	0.001
S_CT	−4.91 ± 5.35	−9.5 ± 5.31	−5.073	0.000

Data are given as median with 25–75% interquartile range or mean ± standard deviation. *P* < 0.05 was considered statistically significant. NVPAF, nonvalvular paroxysmal atrial fibrillation; LAVmin, left atrial minimum volume; LAVmax, left atrial maximum volume; LAVpreA, volume at onset of left atrial contraction; LAEV, left atrial evacuation volume; LAEF, left atrial ejection fraction; S_R, left atrial reservoir strain; S_CD, left atrial conduit strain; S_CT, left atrial contractile strain.

**Table 3:** Logistic regression analysis results of LAF parameters in patients with nonvalvular paroxysmal atrial fibrillation.

Factors	Coefficients	SE	*P* value	OR	95% CI
LAVmin	0.088	0.056	0.114	1.092	0.979∼1.218
LAVmax	0.009	0.038	0.816	1.009	0.937∼1.086
LAVpreA	−0.071	0.045	0.114	0.931	0.852∼1.017
LAEF	−0.124	0.033	0.000	0.883	0.827∼0.943
S_R	−0.088	0.243	0.047	0.916	0.569∼1.474
S_CD	−0.170	0.248	0.494	0.844	0.519∼1.372
S_CT	−0.158	0.242	0.513	0.854	0.531∼1.371

OR, odds ratio; *P* < 0.05 was considered statistically significant.

**Table 4:** Interobserver and intraobserver variability.

Parameters	Interobserver variability			Intraobserver variability		
	ICC	95% CI	*P* value	ICC	95% CI	*P* value
LAVmin	0.991	0.987–0.998	<0.001	0.991	0.973–0.996	<0.001
LAVmax	0.992	0.990–0.999	<0.001	0.995	0.992–0.999	<0.001
LAVpreA	0.992	0.991–0.998	<0.001	0.995	0.993–0.999	<0.001
LAEV	0.991	0.983–0.997	<0.001	0.986	0.967–0.996	<0.001
LAEF	0.992	0.974–0.996	<0.001	0.978	0.940–0.990	<0.001
S_R	0.993	0.982–0.997	<0.001	0.984	0.965–0.994	<0.001
S_CD	0.991	0.974–0.997	<0.001	0.977	0.949–0.992	<0.001
S_CT	0.993	0.988–0.998	<0.001	0.989	0.972–0.995	<0.001

ICC, intraocular correlation coefficient; CI, confidence interval.

**Table 5:** Kaplan–Meier univariate analysis.

Factors	Classification	Median survival time (days)	Log rank chi-square	*P* value
Therapy method	Radiofrequency ablation	490	9.414	0.002
	Nonradiofrequency ablation	349		

Embolism risk	Low risk	434	7.970	0.005
	High risk	310		

Low risk, CHA2DS2-VASc scores of <2; High risk, CHA2DS2-VASc scores of ≥2; CHA2DS2-VASc, CHA2DS2-VASc, cardiac failure, high blood pressure, age of 65–74 years (doubled), diabetes mellitus, stroke or temporary ischemic attack (TIA), and sex group (female).

**Table 6:** Cox univariate and multivariate analyses.

Factors	Univariate		Multivariate	
	HR (95% CI)	*P*	HR (95% CI)	*P*
S_R	0.829 (0.764–0.901)	0.000	0.890 (0.811–0.978)	0.003
LAEF	0.890 (0.843–0.939)	0.000	0.912 (0.859–0.969)	0.015
Therapy method	—	0.002	0.227 (0.046–1.132)	0.071
Thromboembolic risk	—	0.005	1.231 (0.489–3.099)	0.658

S_R, LA reservoir strain; LAEF, left atrial emptying fraction; HR, risk ratio.

## Data Availability

The data used to support the findings of this study are included within the article.

## References

[1] January C. T., Wann L. S., Calkins H. (2019). 2019 AHA/ACC/HRS focused update of the 2014 AHA/ACC/HRS guideline for the management of patients with atrial fibrillation: a report of the American college of Cardiology/American heart association task force on clinical practice guidelines and the heart rhythm society in collaboration with the society of thoracic surgeons. *Circulation*.

[2] Verdecchia P., Angeli F., Reboldi G. (2018). Hypertension and atrial fibrillation: doubts and certainties from basic and clinical studies. *Circulation Research*.

[3] Tousoulis D. (2019). Biomarkers in atrial fibrillation; from pathophysiology to diagnosis and treatment. *Current Medicinal Chemistry*.

[4] Bhatt H. V., Fischer G. W. (2015). Atrial fibrillation: pathophysiology and therapeutic options. *Journal of Cardiothoracic and Vascular Anesthesia*.

[5] Najib M. Q., Vinales K. L., Vittala S. S., Challa S., Lee H. R., Chaliki H. P. (2012). Predictors for the development of severe tricuspid regurgitation with anatomically normal valve in patients with atrial fibrillation. *Echocardiography*.

[6] Zimetbaum P. (2017). Atrial fibrillation. *Annals of Internal Medicine*.

[7] Edwards J. D., Healey J. S., Fang J., Yip K., Gladstone D. J. (2020). Atrial cardiopathy in the absence of atrial fibrillation increases risk of ischemic stroke, incident atrial fibrillation, and mortality and improves stroke risk prediction. *Journal of the American Heart Association*.

[8] Hindricks G., Potpara T., Dagres N. (2021). ESC Guidelines for the diagnosis and management of atrial fibrillation developed in collaboration with the European Association for Cardio-Thoracic Surgery (EACTS): the Task Force for the diagnosis and management of atrial fibrillation of the European Society of Cardiology (ESC) Developed with the special contribution of the European Heart Rhythm Association (EHRA) of the ESC. *European Heart Journal*.

[9] Sugimoto T., Robinet S., Dulgheru R. (2018). Echocardiographic reference ranges for normal left atrial function parameters: results from the EACVI NORRE study. *European Heart Journal-Cardiovascular Imaging*.

[10] Leung M., Abou R., van Rosendael P. J. (2018). Relation of echocardiographic markers of left atrial fibrosis to atrial fibrillation burden. *The American Journal of Cardiology*.

[11] Habibi M., Zareian M., Ambale Venkatesh B. (2019). Left atrial mechanical function and incident ischemic cerebrovascular events independent of AF: insights from the MESA study. *Journal of the American College of Cardiology: Cardiovascular Imaging*.

[12] Liao Y. C., Liao J. N., Lo L. W. (2017). Left atrial size and left ventricular end-systolic dimension predict the progression of paroxysmal atrial fibrillation after catheter ablation. *Journal of Cardiovascular Electrophysiology*.

[13] Dissabandara T., Lin K., Forwood M., Sun J. (2023). Validating real-time three-dimensional echocardiography against cardiac magnetic resonance, for the determination of ventricular mass, volume and ejection fraction: a meta-analysis. *Clinical Research in Cardiology*.

[14] Heijman J., Voigt N., Nattel S., Dobrev D. (2014). Cellular and molecular electrophysiology of atrial fibrillation initiation, maintenance, and progression. *Circulation Research*.

[15] Ding W. Y., Khan A. A., Gupta D., Lip G. Y. H. (2019). Short-term outcomes in newly diagnosed atrial fibrillation and chronic kidney disease: how important is ethnicity?. *Journal of the American Heart Association*.

[16] Bao L., Cheng L., Gao X. (2022). Left atrial morpho-functional remodeling in atrial fibrillation assessed by three dimensional speckle tracking echocardiography and its value in atrial fibrillation screening. *Cardiovascular Ultrasound*.

[17] Donal E., Lip G. Y., Galderisi M. (2016). EACVI/EHRA Expert Consensus Document on the role of multi-modality imaging for the evaluation of patients with atrial fibrillation. *Eur Heart J Cardiovasc Imaging*.

[18] Jiang F., Chen Y., Wu L. (2021). Left heart function evaluation of patients with essential hypertension and paroxysmal atrial fibrillation by two-dimensional speckle tracking imaging combined with real-time three-dimensional ultrasound imaging. *Journal of Thoracic Disease*.

[19] Bao L., Cheng L., Bao L. (2022). Left atrial function and its correlation with left ventricular filling pressure in patients with paroxysmal atrial fibrillation: a case-control study. *[J] Fudan Journal (Medical Edition)*.

[20] Chen L., Zhang C., Wang J. (2021). Left atrial strain measured by 4D Auto LAQ echocardiography is significantly correlated with high risk of thromboembolism in patients with non-valvular atrial fibrillation. *Quantitative Imaging in Medicine and Surgery*.

[21] Providência R., Trigo J., Paiva L., Barra S. (2013). The role of echocardiography in thromboembolic risk assessment of patients with nonvalvular atrial fibrillation. *Journal of the American Society of Echocardiography*.

[22] Habibi M., Lima J. A., Khurram I. M. (2015). Association of left atrial function and left atrial enhancement in patients with atrial fibrillation: cardiac magnetic resonance study. *Circulation: Cardiovascular Imaging*.

[23] Liu H., Chen L., Song Y. (2023). Use of left atrial automated functional myocardial imaging to identify patients with paroxysmal atrial fibrillation at high risk of stroke. *Quantitative Imaging in Medicine and Surgery*.

